# Quantitative Understanding of Advanced Novel Imaging Techniques for Fasciitis and Biosignature Yield (Quantify): Protocol for a Cross-Sectional Diagnostic Study

**DOI:** 10.2196/87613

**Published:** 2026-02-11

**Authors:** Zahra Amerian, Timothy Fleagle, Utsav Tuladhar, Rachel Watson, Micah Wong, Barbara Van Gorp, Brian J Smith, Michael Richards, Mederic Hall, Joe Donnelly, Jessica Danielson, Renata Vidal Leao, David Stanley, Vincent Magnotta, James H Holmes, Kathleen Sluka, Ruth L Chimenti

**Affiliations:** 1 Department of Physical Therapy and Rehabilitation Science University of Iowa Iowa City, IA United States; 2 Department of Biomedical Engineering Rochester Institute of Technology Rochester, NY United States; 3 Department of Biostatistics College of Public Health University of Iowa Iowa City, IA United States; 4 Iowa Orthopedics, Sports Medicine and Rehabilitations University of Iowa Iowa City, IA United States; 5 Department of Orthopedics and Rehabilitation University of Iowa Iowa City, IA United States; 6 Physical Therapy Program College of Rehabilitative Sciences University of St Augustine for Health Sciences Coral Gables, FL United States; 7 Institute for Clinical and Translational Science, University of Iowa Iowa City, IA United States; 8 Department of Radiology Carver College of Medicine University of Iowa Iowa City, IA United States; 9 Department of Neuroscience and Pharmacology University of Iowa Iowa City, IA United States

**Keywords:** fasciitis, plantar, Achilles tendon, biomarker, myofascial, ultrasound imaging, magnetic resonance imaging, pain

## Abstract

**Background:**

Myofascial pain remains an underdiagnosed contributor to musculoskeletal pain conditions, including plantar heel pain, which is the most common source of foot pain. The current standard for diagnosing myofascial pain is a clinical examination using manual palpation. However, this approach lacks quantitative thresholds for precise assessment of myofascial pain, highlighting the need for validated biomarkers.

**Objective:**

This protocol describes the development of a diagnostic imaging biosignature of myofascial pain using both ultrasound and magnetic resonance imaging to differentiate individuals with plantar heel pain from those with other kinds of foot pain and matched pain-free controls. The study will also explore whether diagnostic accuracy is enhanced by creating a composite biosignature that includes psychological factors.

**Methods:**

In this cross-sectional study, 100 participants will be recruited: 50 with plantar heel pain, 25 with insertional Achilles tendinopathy, and 25 pain-free controls. Participants will undergo a clinical examination of 5 calf and foot muscles to identify sites of abnormal myofascial tissue. The primary imaging outcomes will capture biochemical properties (T1ρ of muscle and fascia), biomechanical properties (shear wave speed of the muscle, shear strain of the plantar fascia during passive movement), and structural profile (fat fraction of the muscle, thickness of the plantar fascia). Patient-reported outcomes will include the National Institutes of Health’s Helping to End Addiction Long-term (HEAL) Initiative Common Data Elements and additional psychological measures.

**Results:**

This study is supported by grant R61AT012275 from the National Center for Complementary and Integrative Health and the National Institute of Neurological Disorders and Stroke, awarded in September 2024. Participant enrollment began in May 2025. As of November 2025, a total of 55 participants have been enrolled. Enrollment is expected to conclude no later than July 2026. The anticipated study completion date is August 2026. Data will be shared within 1 year of completing the study or upon publication, whichever occurs first.

**Conclusions:**

This protocol provides novel mechanistic insight into myofascial pain through advanced imaging techniques, offering a biopsychosocial framework for improving the diagnosis and treatment of plantar heel pain and related conditions. We anticipate that combining imaging and psychosocial measures will improve the diagnostic accuracy of the biosignature and provide a more comprehensive understanding of myofascial pain.

**Trial Registration:**

ClinicalTrials.gov NCT06803056; https://clinicaltrials.gov/study/NCT06803056; OSF Registries osf.io/nxqfj; https://osf.io/nxqfj

**International Registered Report Identifier (IRRID):**

DERR1-10.2196/87613

## Introduction

Plantar heel pain, the most common source of foot pain, affects approximately 1 out of every 10 adults and accounts for approximately 2 million medical visits annually in the United States [1,2]. Individuals with plantar heel pain frequently experience reduced quality of life and diminished satisfaction with activities of daily living [3,4]. Plantar heel pain is characterized by pain along the sole, most noticeable during initial steps following a period of inactivity and during prolonged weight-bearing activities [5]. The term “plantar fasciitis” is often used to describe this condition and suggests inflammation as the main cause of heel pain. However, the broader term “plantar heel pain” is preferred, reflecting the multifactorial nature of the underlying causes—including myofascial changes, adjacent tissue pathology, and psychosocial factors [5-9].

One potentially underrecognized component of plantar heel pain is myofascial pain, which is characterized by the presence of trigger points in the calf and foot muscles that can reproduce the individual’s pain [10,11]. Trigger points are defined as hypersensitive areas within a tight band of skeletal muscle that elicit local or referred pain when compressed, contracted, or stretched. The current standard for diagnosing myofascial pain relies on a clinical examination using manual palpation [12,13], which lacks objectivity and quantitative precision. Identifying a diagnostic imaging biomarker [14] for trigger points could significantly improve the accuracy of diagnosing myofascial pain and inform targeted treatment strategies.

Imaging is a powerful, yet underused, diagnostic tool that can be used to identify muscles with trigger points [15]. Ultrasound and magnetic resonance imaging (MRI) are both commonly used clinically to evaluate soft tissues [16-18]. Ultrasound is a noninvasive imaging technique with minimal contraindications that provides real-time assessments of soft tissue, including muscles and fascia [19]. In particular, ultrasound shear wave elastography (SWE) demonstrates high diagnostic accuracy in capturing abnormal biomechanical properties of myofascial tissue [15]. MRI is often regarded as the gold standard for evaluating soft tissues due to its ability to provide superior contrast, high resolution, and multiplanar images [18]. Previous studies have shown promise using T1 signal intensity and T2 mapping to detect structural alterations in trapezius muscles in women with migraines [20-22]. To build on previous foundational work, further research is needed to examine the diagnostic capabilities of both ultrasound and MRI within the same sample and to expand imaging approaches for identifying trigger points in the foot and ankle.

The primary aim of this proposed study is to develop a diagnostic imaging biosignature of myofascial pain to differentiate individuals with plantar heel pain from those with other kinds of foot pain (specifically insertional Achilles tendinopathy) and from matched pain-free controls. We hypothesize that a diagnostic biosignature, which uses both advanced ultrasound and MRI to quantify biochemical (muscle and fascia T1ρ), biomechanical (muscle SWE and fascia strain), and structural (muscle fat fraction and fascia thickness) tissue properties, will more accurately identify myofascial pain in the foot and ankle than a single imaging biomarker alone. We will also explore the potential of composite biosignatures that combine multiple imaging biomarkers with psychological factors, such as kinesiophobia, to provide a more comprehensive assessment. The development of a diagnostic biosignature may provide novel mechanistic insights into myofascial pain and offer a framework to facilitate the diagnosis of myofascial pain contributing to plantar heel pain.

## Methods

### Participants

This cross-sectional study will enroll a total of 100 participants, including 50 individuals diagnosed with plantar heel pain, along with 2 control groups. The first control group will consist of 25 individuals with insertional Achilles tendinopathy, for whom targeted myofascial treatments are not considered first-line interventions [23]. The second control group will include 25 pain-free individuals with a similar age, BMI, and sex distribution as the plantar heel pain and insertional Achilles tendinopathy groups. To ensure balanced representation, enrollment will be structured so that neither sex comprises more than 60% of each group. Eligibility and group assignment will be based on the inclusion and exclusion criteria outlined in Textbox 1. Screening for study eligibility will occur in three phases: (1) an online screening form, (2) a video visit with a physical therapist, and (3) an in-person clinical examination by a physical therapist. Individuals who provide informed consent and meet all criteria will be enrolled in this study.

**Eligibility criteria.**

**Inclusion criteria**

- For the plantar heel pain and insertional Achilles tendinopathy group:
- Clinical diagnosis of plantar heel pain [5] or insertional Achilles tendinopathy [23].
- Duration of pain ≥3 months.
- Severity of pain ≥3/10.

**Exclusion criteria**

- For all groups:
- Age <18 years.
- History of invasive procedures to the foot or ankle on the side of interest.
- History of lower extremity injections, dry needling, extracorporeal pulse activation treatment, or extracorporeal shockwave therapy within the past 3 months on the side of interest.
- Contraindications for MRI (eg, non–magnetic resonance–compatible implanted devices, claustrophobia, inability to remain still comfortably for 1 hour in a supine position [24])
- Clinically unstable medical or psychiatric issues.
- Comorbidities associated with changes in musculoskeletal imaging, including rheumatologic and inflammatory conditions that directly impact the musculoskeletal system (eg, rheumatoid arthritis, spondyloarthropathy, and gout), osteoarthritis of the foot or ankle, diabetes, neuromuscular diseases (eg, Charcot-Marie-Tooth [CMT] disease), familial hypercholesterolemia, history of foot or ankle fracture, neoplasms involving the foot, plantar fibromatosis, or recent (within the past 1 year) infection of the foot or ankle (eg, infectious fasciitis and calcaneal osteomyelitis).
- For the plantar heel pain and insertional Achilles tendinopathy group:
- Other sources of heel pain (eg, tarsal tunnel syndrome, peripheral neuropathy, lumbar radiculopathy, calcaneal stress fracture, fibromyalgia, Morton’s neuroma, or Baxter neuropathy).
- Additional exclusion criteria for insertional Achilles tendinopathy group: co-occurring plantar heel pain and Achilles tendinopathy.
- For the pain-free control group:
- Persistent or recurrent leg pain in the past 6 months.

### Data Collection Procedures

#### Overview

**Figure 1:**
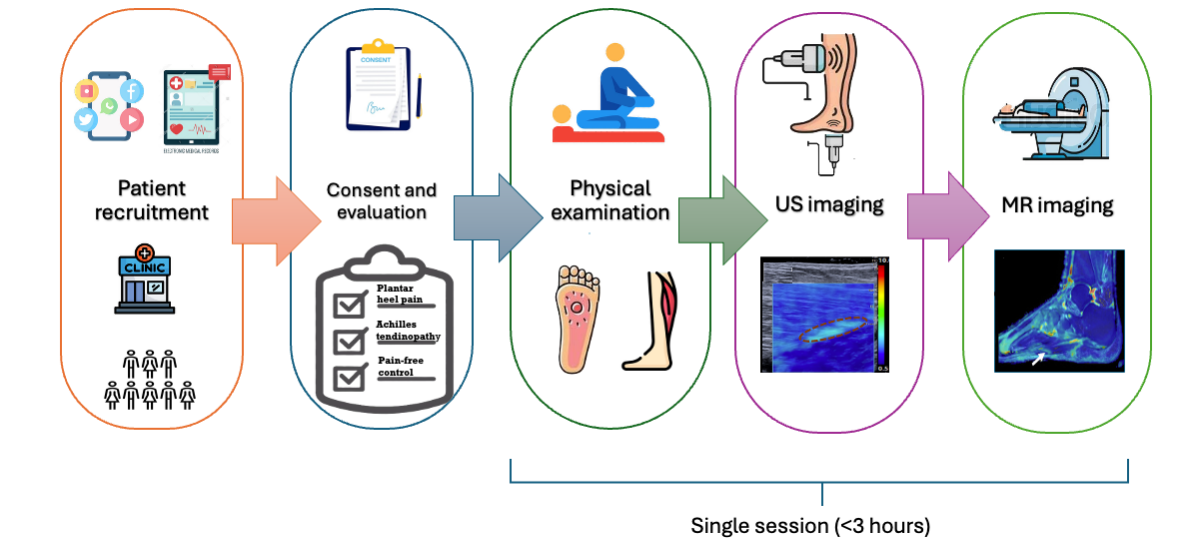
Study flow diagram. This diagram outlines the sequential study procedures, starting with participant recruitment, consent, and eligibility assessment. It proceeds through a clinical examination of the calf and foot muscles to identify trigger points. Following the clinical assessment, participants undergo ultrasound and magnetic resonance imaging (MRI) with minimal delay to ensure spatial correspondence of identified trigger points across imaging modalities. The entire study visit lasts approximately 3 hours in a single session. MR: magnetic resonance; US: ultrasound.

This cross-sectional study will include 3 main components of data collection: a clinical evaluation, ultrasound imaging, and MRI (Figure 1).

#### Clinical Examination

The clinical examination will be performed by a physical therapist with advanced training in dry needling for myofascial pain and will include (1) movement tasks, (2) trigger point identification, and (3) pressure pain threshold testing. In addition, participants will complete a series of patient-reported outcome measures. These outcomes align with the HEAL Common Data Elements and are supplemented by additional psychological surveys, as well as foot- and ankle-specific pain and function outcomes [25] (Table 1).

**Table 1:** Patient-reported outcome measures.

Outcome							Description		Psychometrics
**Pain**									
		PEG^a^				A 3-item instrument that evaluates pain intensity and its impact on enjoyment and general activity. Each item is scored from 0 to 10, and the 3 scores are averaged to yield a total PEG score, with higher values indicating greater pain and interference [26].		Internal consistency: Cronbach α=0.73 Validity: r=0.60-0.89 (in chronic musculoskeletal pain populations)	
	PROMIS^b^-Pain Interference Short Form				Assesses pain interference, which quantifies the self-reported impact of pain on social, cognitive, emotional, physical, and recreational domains. This instrument yields raw scores from 6 to 30, based on 6 items scored from 1 (“not at all”) to 5 (“very much”). Scores are converted to T-scores (mean 50, SD 10; range ~30 to over 80) standardized to the US general population. T-scores below 55 are within normal limits, 55-60 suggest mild, 60-70 moderate interference, and scores >70 reflect severe interference [27-29].				Internal consistency: Cronbach α=0.96-0.99 Test-retest reliability: ICC^c^=0.85 Convergent validity with the Brief Pain Inventory: r=0.80-0.85
Physical function									
	PROMIS^b^-Physical Function Short Form 6b					Assesses the broader influence of pain on life domains, with raw scores ranging from 6 to 30; each item is scored from 1 (“unable to do”) to 5 (“without any difficulty”). Converted T-scores (mean 50, SD 10) range approximately from 20 to >80. T-scores >45 are within normal limits, scores from 40 to 45 suggest mild limitation, scores from 30 to 40 indicate moderate limitation, and scores <30 reflect severe limitation [29-31].		Internal consistency: Cronbach α=0.96 for person and 0.99 for item Construct validity: r=0.657 (in patients undergoing foot and ankle surgery)	
	FAAM^d^				Provides targeted assessment of foot and ankle function across two subscales: (1) activities of daily living (ADL), which includes 21 items scored from 0 (“unable to do”) to 4 (“no difficulty”), with a total score range of 0-84; and (2) sports, which includes 8 items with a total score range of 0-32. Scores are converted to percentages, with higher percentages reflecting better function [32].				Test-retest reliability: ICC=0.89 for the ADL; ICC=0.87 for the Sports. Correlations with the SF-36 physical function subscale: r=0.84-0.78 and physical component summary score: r=0.78-0.80
	VISA-A Sedentary^e^			Evaluates the severity of Achilles tendinopathy in sedentary individuals. It consists of 8 variably scored items addressing symptoms and activity impact, with a total score ranging from 0 to 100. A score of 100 indicates full function without symptoms, whereas scores <70 suggest notable impairment [33].					Test–retest reliability: ICC=0.991 for symptom and ICC=0.999 for activity Internal consistency: Cronbach α=0.72 Construct validity: r=0.42 for symptoms; r=0.40 for activity
**Psychological factors**									
	PCS-6^f^		A short form of the Pain Catastrophizing Scale that measures catastrophic thinking about pain. It consists of 6 items across 3 subscales—2 items each for “rumination,” “helplessness,” and “magnification”—each scored from 0 to 4, yielding a total score of 0-24, with higher scores indicating greater catastrophic thinking [34,35].					Internal consistency: Cronbach α=0.88 Correlation with the full 13-item PCS: r=0.94 in individuals with chronic pain conditions	
	TSK-11^g^					Measures fear of movement using 11 items from 1 (“strongly disagree”) to 4 (“strongly agree”), yielding a total score of 11-44. Scores <22 indicate minimal fear, 23-28 low fear, 29-35 moderate fear, and ≥36 high fear [36,37].		Convergent validity: association with pain catastrophizing, severity of expected pain with movement, and willingness to complete tendon loading exercises in individuals with Achilles tendinopathy Test-retest reliability: ICC=0.81 (in individuals with back pain)	
	PHQ-2^h^		Screens for depression over the past 2 weeks using 2 items scored from 0 (“not at all”) to 3 (“nearly every day”), yielding a total range of 0-6. Scores of 0-2 suggest minimal or no depression, while scores ≥3 indicate possible depression requiring further evaluation [36].					Sensitivity: 83% Specificity: 92%	
	PROMIS^b^ Sleep Disturbance					Evaluates sleep quality and disturbances across 8 items, each scored from 1 (“not at all”) to 5 (“very much”), yielding raw scores of 8-40. Scores are converted to T-scores (mean 50, SD 10), ranging approximately from 20 to >80. T-scores of 55-60 indicate mild sleep disturbance, 60-70 suggest moderate disturbance, and >70 reflect severe sleep disturbance [37,38].		Internal consistency: Cronbach α=0.96 Convergent validity with the Pittsburgh Sleep Quality Index: r=0.85 Test-retest reliability: ICC=0.62-0.71 (in fibromyalgia populations)	
	PROMIS^b^ Self-Efficacy for Managing Symptoms 8a			Assess the subject’s level of confidence in managing their symptoms in different settings (home, public place, and an unfamiliar place) and in keeping their symptoms from interfering with work, sleep, relationships, or recreational activities. Each item scored from 1 (“not at all confident”) to 5 (“very confident”), yielding raw scores from 8 to 40. Converted T-scores below 30 suggest very low, 30-40 low, 40-60 average, 60-70 high, and 70-80 reflect very high self-efficacy [39].					Internal consistency: Cronbach α=0.92 Correlations with Chronic Pain Self-Efficacy Scale: r=0.74 (in patients with chronic conditions)
	GAD-2^i^			Screens for generalized anxiety disorder by asking about the frequency of feeling nervous and unable to control worrying over the past 2 weeks. It uses 2 items, each scored from 0 (“not at all”) to 3 (“nearly every day”), totaling 0 to 6. Scores of 0 to 2 indicate minimal or no anxiety, while scores of 3 or higher suggest possible anxiety requiring further assessment [40].					Sensitivity: 76% Specificity: 81% Internal consistency: Cronbach α=0.89
**Comorbidities**									
	TAPS-1^j^			Screens for substance use in the past 12 months. Scores range from 0 to 4 (0=“never” and 4=“daily or almost daily”) for each substance category, with higher scores indicating more frequent use [41].					Sensitivity: 81%-96% Specificity: 78%-91%
	CCI^k^					Predicts 10-year mortality for patients with multiple comorbidities based on 19 weighted conditions, scored from 1 to 6 points, with a total range of 0 to >37. Higher scores correspond to a greater comorbidity burden [42-44].		Test-retest reliability: ICC=0.94 (for self-report data compared to chart reviews) Inter-rater reliability: ICC=0.80	
	WPI^l^					Assesses pain distribution across 19 body areas, assigning a score of 0 if there is no pain and 1 if pain is present in each area. A total score of 7 or more typically indicates widespread pain. The SSS^m^ scale evaluates symptom severity in 2 components: the first assesses fatigue, unrefreshing sleep, and cognitive difficulties, while the second derives a general symptom severity score based on the number of symptoms reported from a list of 41 symptoms. Patients receive a score of 0 for no symptoms, 1 for 1-10 symptoms, 2 for 11-24 symptoms, and 3 for 25 or more symptoms. The total SSS score ranges from 0 to 12, calculated by summing the scores from the first component (range 0-9) and the second (range 0-3) [45].		Internal consistency: Cronbach α=0.34 for the WPI, 0.83 for the SS, and 0.82 for the combined use of both tools. Sensitivity: 100% and Specificity: 81% for WPI Sensitivity: 89.2% and Specificity: 83.8% for SSS	

^a^PEG: Pain, Enjoyment, General Activity.

^b^PROMIS: Patient-Reported Outcomes Measurement Information System.

^c^ICC: intraclass correlation coefficient.

^d^FAAM: Foot and Ankle Ability Measure.

^e^VISA-A Sedentary: Victorian Institute of Sport Assessment–Achilles (sedentary version).

^f^PCS-6: Pain Catastrophizing Scale–6.

^g^TSK-11: Tampa Scale for Kinesiophobia–11.

^h^PHQ-2: Patient Health Questionnaire–2.

^i^GAD-2: Generalized Anxiety Disorder–2.

^j^TAPS-1: Tobacco, Alcohol, Prescription medication, and other Substance use.

^k^CCI: Charlson Comorbidity Index.

^l^WPI: Widespread Pain Index.

^m^SSS: Symptoms Severity Scale.

#### Movement Tasks

Pain intensity will be reported using the numeric rating scale (NRS), where 0 indicates no pain, and 10 indicates the worst pain imaginable [46,47]. The NRS has been shown to have excellent test-retest reliability (intraclass correlation coefficient=0.95) and concurrent validity with good-to-excellent correlation with the visual analog scale (r=0.941) [48]. Participants will rate their level of pain at rest. In addition, they will report their heel pain (plantar fascia or Achilles tendon, depending on group assignment) during the following three movement tasks: (1) the “Windlass test” will be performed in a standing position with passive extension of the participant’s great toe to the end range while bearing weight on the affected foot. A positive test will be indicated by reproduction or exacerbation of plantar heel pain [5,49]. The weight-bearing Windlass test demonstrates 100% specificity and 31.8% sensitivity [49]; (2) for the “single-leg heel raise test,” participants will stand on the affected leg with their fingertips on a wall in front of them to provide balance. Keeping the knee straight, they will then lift and lower their heel as high as possible. They will perform as many repetitions as they can while keeping pace with a metronome beat played from a phone application (60 Hz). The task will continue until muscle fatigue or symptom exacerbation prevents further movement. The test will be discontinued if the participant is unable to maintain proper technique, such as bending the knee, flexing the trunk, or failing to keep pace with the metronome. If a participant is unable to perform single-limb heel raises, the test will be modified to a double-limb heel raise, instructing them to shift as much weight as possible onto the involved side [5,50,51]; and (3) the “single-leg drop landing test” will be conducted by asking participants to step onto a platform while leading with their affected leg and then step back down, landing only on the affected foot [52]. If this movement does not provoke pain or symptoms, participants will be instructed to add a vertical jump immediately after landing on the involved foot [53].

#### Trigger Point Identification

Participants will lie in the prone position with knees flexed at 15° and ankles in their naturally relaxed plantarflexed posture. A physical therapist will examine 5 muscles: 3 in the calf (medial gastrocnemius, lateral gastrocnemius, and medial soleus) and 2 in the foot (abductor hallucis and flexor digitorum brevis). To facilitate consistency in the examination and imaging, the assessment will be limited to the following muscle zones, which Simons [54] has described as common trigger point locations.

Calf zones will be centered at 50% of the leg length measured from the fibular head, with the gastrocnemius zone extended proximally by 40% of the leg length, while the soleus zone will be extended distally by 30% of the leg length. For foot muscles, the abductor hallucis zone will be centered along a vertical line from the anterior medial malleolus, with medial and lateral boundaries extending 5% of the leg length [55]. The flexor digitorum brevis zone will begin 2 cm distal to the medial calcaneal tubercle and span 10% of the leg length distally. Leg length will be measured from the fibular head to the lateral malleolus. Using standardized zones based on leg length ensures that similar regions of the muscle belly are captured and helps ensure that the distance between the most proximal and distal trigger points is no greater than 20 cm, which corresponds to the MRI field of view.

The physical therapist will palpate within the 5 zones to determine if a trigger point is present and mark the tautest band using a waterproof marker in a cross shape in each zone. This approach allows for the same locations to be assessed with ultrasound imaging and MRI. Trigger point identification will follow the gold standard diagnostic criteria established by Simons [54], who defines a trigger point as a taut band of skeletal muscle that is tender to palpation. The physical therapist will apply constant pressure with palpation for 5 seconds on the most tender taut band in each of the 5 zones, with a 20-second rest between each spot. Participants will be asked whether the pressure reproduces the same heel pain [54] and whether the pain is felt locally at the site of palpation or referred to another area [10] at each site. For comparison of imaging outcomes between trigger points and healthy tissue, at least 1 of the 5 marks will indicate healthy tissue using the same cross shape. Imaging researchers will remain blinded to the trigger point versus healthy tissue identification to ensure unbiased image acquisition.

#### Pressure Pain Threshold Test

Following the completion of ultrasound and MRI, a pressure pain threshold test will be conducted using a pressure algometer (Somedic Algometer Type II) on each of the 5 previously marked locations on the leg and foot [56-60]. The algometer will be positioned perpendicularly to these sites, and pressure will be consistently applied downward at a constant rate of 50 kPa/s [61,62] using a 1-cm^2^ round rubber tip probe. Participants will be instructed to press a handheld button at the moment they first perceive the pressure sensation as painful, defined as anything greater than 0/10 on the NRS [63]. Three measurements will be taken per site, with at least 30 seconds between repetitions to reduce temporal summation effects. Participants with even and odd ID numbers will be tested in different sequences of muscle groups to minimize order effects.

### Ultrasound Imaging

Ultrasound imaging will be performed by a physical therapist experienced in ultrasound imaging of the foot and ankle. To provide consistent positioning across the ultrasound and MRI scans, the participant will be in the prone position with the knee flexed to 15° and a strap around the ball of the foot applying slight pressure to maintain the ankle in a slightly plantarflexed position (approximately 20°). This ankle position replicates the 1.5-inch lift under the heel provided during the MRI. Ultrasound imaging will be acquired using the ACUSON Sequoia Ultrasound System (Siemens Healthineers). For SWE, a 10L4 linear array transducer (2.9-9.9 MHz) will be positioned parallel with the muscle fibers, centered over the 5 marks identified during the clinical examination. Because the plantar fascia lies immediately superficial to the flexor digitorum brevis, its shear wave speed will also be captured. In addition, as an exploratory measure, the transducer will also be positioned parallel with the Achilles tendon to capture the region immediately proximal to the calcaneus. A standoff pad will be used for Achilles tendon and abductor hallucis muscle imaging to allow for a similar imaging depth as the calf muscles, which have a greater amount of superficial adipose tissue. Three images per location will be collected. To quantify shear wave velocity, a custom MATLAB code will be used to allow for the selection of the tissue region of interest, with analysts blinded to the overlay of the shear wave velocity map. The average of the 3 replicates will be used to estimate a representative shear wave velocity for that tissue [64].

The thickness of the plantar fascia will be measured using the maximum of 2 B-mode images, a long-axis and a short-axis view, at the insertion to the calcaneus. For the plantar fascia strain, an 18L6 linear array transducer (5.5-18 MHz) will be positioned parallel with the plantar fascia for a long-axis view. A cine loop will be collected while the participant’s great toe is passively extended from neutral to the end range of motion over 5 seconds. Simultaneously, a video of the foot will be captured on a tablet camera placed approximately 1 foot medial to the participant’s ankle, with the foot centered in the video frame and the screen positioned at a 90° angle relative to the floor. This passive range of motion task and imaging protocol will be repeated 3 times. A nonrigid image registration–based strain estimator will be used to measure the accumulated displacement field in the fascia throughout the first toe passive extension task [65]. The displacement estimates will then be used to quantify the average shear and principal strains within the plantar fascia during the passive movement. The magnitude of toe extension will be quantified using Kinovea software (open-source, version 0.9.5; Joan Charmant).

### MRI

To ensure spatial alignment between clinical examination, ultrasound imaging, and MRI, magnetic resonance–visible fiducial markers will be placed over the marked trigger points identified during clinical examination. MRI will be conducted using a 3T whole-body MRI (Signal Premier, GE Healthcare). Scanning will begin using an 8-channel foot-ankle receive RF coil (GE Healthcare, Waukesha) for imaging the foot, followed by a 32-channel flexible blanket array receive RF coil (GE Healthcare) for imaging the calf. Imaging will be performed on the affected side in participants with plantar heel pain and insertional Achilles tendinopathy, and on a single side in pain-free controls, with all participants scanned in the supine position with slight knee flexion and ankle plantar flexion to accommodate a 1.5-inch heel lift between the heel and the RF coil (approximately 20° of plantar flexion).

The imaging will include the following sequences: T2-weighted fast spin-echo sequences for T2 mapping and evaluation of inflammation and cysts [66]; zero echo time proton density sequences to detect bony abnormalities [67,68]; quantitative T1ρ imaging, which provides sensitivity to multiple features such as chemical exchange between extracellular water, macromolecules and pH [69,70]; chemical shift–encoded Iterative Decomposition of water and fat with Echo Asymmetry and Least-squares estimation for T2* and fat fraction quantification to assess tissue composition [71,72]; intravoxel incoherent motion (IVIM) diffusion imaging to evaluate tissue perfusion; and T1-weighted imaging to measure plantar fascia and fat pad thickness.

MRI data will be analyzed using both quantitative and qualitative approaches. Regions of interest (ROI) will be measured based on the fiducial markers corresponding to the locations identified during the clinical examination. Water and fat images will be generated using the Iterative Decomposition of water and fat with Echo Asymmetry and Least-squares estimation (IDEAL) for the separation of fat and water species [71, 72], Specifically, these will be produced using the vendor supplied chemical shift–encoded 3D gradient-echo sequence (IDEAL-IQ, GE Healthcare, WI, USA).

The IDEAL-IQ gradient-echo images will be used for marker identification and tissue segmentation, and will serve as the reference images for spatially registering the other MRI images to a common image-space. The ROIs will be delineated using the IDEAL-IQ images, guided by the locations of the fiducial markers that are superficial to the trigger points. Detailed MRI scan parameters for the foot and lower leg, respectively, are provided in Tables S1 and S2. The corresponding ROIs will then be used to measure T1ρ, T2*, perfusion fraction, and pseudo-perfusion from IVIM, as well as fat–water fractions. Measurements from each ROI will include the mean, median, and SD. Quantitative maps of fat–water fraction and T2* will be automatically generated by the scanner using the IDEAL-IQ sequence, while voxel-wise parametric maps for T1ρ, T2, and IVIM will be produced using in-house software.

Qualitative image assessments will be performed by a radiologist to exclude differential diagnoses for plantar heel pain and to confirm accurate identification of leg and foot muscles.

### Statistical Analysis

#### Data Analysis

In the statistical analysis, quantitative measures will be summarized with means, SDs, and quantiles, and categorical measures, including the presence of trigger points (trigger points vs healthy tissue), will be summarized with counts and percentages. Data will be described for 3 calf muscles (medial gastrocnemius, lateral gastrocnemius, and medial soleus) and 2 foot muscles (abductor hallucis and flexor digitorum brevis).

The primary study aim is to examine the diagnostic performance of 20 candidate biomarkers (biomechanical, biochemical, and structural) in distinguishing between tissue with and without myofascial pain. Performance will be quantified with the area under the receiver operating characteristic curve (ROC AUC), pooled over the 5 muscle groups assessed on each participant for imaged metrics and trigger points. Five-fold cross-validation will be used to estimate predictive performance, and bootstrap resampling applied to obtain 95% CIs. Participants will be treated as the sampling units in cluster cross-validation and bootstrap sampling algorithms to account for repeated muscle measurements in each participant. To identify the most promising biomarkers and account for multiple comparisons, false discovery rates will be computed with the method of Benjamini and Hochberg to rank the biomarkers in order of diagnostic performance [73]. Up to 5 top-performing biomarkers will be selected to constitute a single biosignature to achieve a target AUC >0.7, sensitivity >60%, and specificity >60%.

In an exploratory aim, composite biosignatures will be developed from imaging biomarkers, patient demographics, and psychological factors. Traditional statistical and machine learning techniques will be used. Logistic regression models, fit with the method of general estimating equations, will be used specifically to test for interactions between imaging biomarkers and sex. Composite models will be built from the full set of predictors using stepwise variable selection as well as the machine learning methods of Lasso, gradient boosting, and random forests. The latter 2 methods have the distinction of performing automated encoding of nonlinear and interaction effects. ROC AUC will be estimated as the measure of diagnostic performance for each model, as described for the primary aim. In addition, variable importance will be reported to quantify the relative contributions of predictor variables, and partial dependence plots will be constructed to show the functional forms of their effects.

#### Sample Size

The target sample size of 50 individuals with plantar heel pain, 25 individuals with insertional Achilles tendinopathy, and 25 pain-free controls was chosen to provide a 95% CI half-width of 0.10 for an AUC of 0.70. This calculation assumes 5 muscle measurements for each participant, an intraclass correlation coefficient of 0.5 among them, and an overall active trigger point prevalence of 36% in individuals with plantar heel pain [10,74]. Further assuming that the 20 candidate biomarkers have AUCs uniformly distributed from 0.5 to 0.8, the study will have at least 85% power to obtain a biosignature meeting the desired AUC threshold.

### Alterations to Study After Initiation

To minimize missing data and maximize data quality, we have made some changes to our study protocol since study initiation. For trigger point identification, we added the step of checking to ensure that the maximum distance between the most proximal and the most distal trigger point is no more than 20 cm, to ensure that they are captured within the magnetic resonance field of view. Also, we adjusted the relative proportions of the regions of ROIs for the gastrocnemius and soleus muscles to minimize inclusion of the gastrocnemius within the soleus ROI during ultrasound imaging. For shear wave ultrasound imaging, we added the use of a gel pad over the abductor hallucis muscle to have a more equivalent imaging depth as the muscles that have a thicker superficial adipose tissue layer. For MRI, we similarly updated the slice number to routinely cover 20 cm in the calf. If global fat-water swaps are encountered, we reduce the readout matrix to 160 to reduce the first echo time, as well as reduce the phase encode number to 160 to maintain consistent voxel dimensions. We also added a B1 mapping sequence to monitor and reduce sensitivity to B1 variations [75]. ROI segmentation was updated to better accommodate oblique scan orientations and data resampling based on registration to the 3D Iterative Decomposition of water and fat with Echo Asymmetry and Least-squares estimation acquisitions. The length of the ROIs was consistent with the diameter of the fiducial markers (1.5 cm).

### Ethical Considerations

This study was preregistered on ClinicalTrials.gov (NCT06803056) and the Open Science Framework (https://osf.io/nxqfj). Ethical approval (IRB 202409110) was obtained from the Biomedical Research Committee (IRB-01) at the University of Iowa. Informed consent will be obtained from all participants before enrollment, and the potential risks and benefits of the study will be clearly explained. Participants will receive compensation for their involvement. All study data will be securely stored in REDCap, a password-protected database hosted by the University of Iowa College of Medicine. The REDCap platform is managed and supported by the Biomedical Informatics Group in the Institute for Clinical and Translational Science at the University of Iowa. REDCap is HIPAA (Health Insurance Portability and Accountability Act)- and 21 CFR 11–compliant, which provides the necessary data security for the proposed clinical trial.

## Results

This study is supported by a grant R61AT012275 from the National Center for Complementary and Integrative Health and the National Institute of Neurological Disorders and Stroke, awarded in September 2024. Participant enrollment began in May 2025. As of November 2025, a total of 55 participants have been enrolled. Enrollment is expected to conclude by no later than July 2026. The anticipated study completion date is August 2026. Data will be shared within 1 year of study completion or upon publication, whichever occurs first.

## Discussion

### Principal Findings

This study is designed to develop a diagnostic imaging biosignature for myofascial pain that differentiates individuals with plantar heel pain from those with other foot pain, such as insertional Achilles tendinopathy, and from pain-free controls. We anticipate that combining imaging and psychosocial measures will improve diagnostic accuracy and provide a more comprehensive understanding of myofascial pain.

### Comparison With Previous Work

The novelty of this study is supported by a multimodal approach, integrating ultrasound and MRI techniques to provide complementary insights into myofascial structures. Despite MRI being considered the gold standard for evaluating soft tissues due to its ability to provide high-resolution detail and multiplanar assessments, its application in myofascial pain research has been limited [15,18]. Previous studies analyzed either T1 signal intensity or T2 mapping, suggesting the promise of these techniques to detect the altered structure of the trapezius muscle in the migraine population [20-22].

### Multimodal Imaging and Biosignature Development

Building on this foundational work, our study will use multiple advanced quantitative MRI techniques, including T1ρ, to capture a broader range of biochemical properties associated with myofascial pain. In addition, the inclusion of matched pain-free controls and individuals with insertional Achilles tendinopathy, a common differential diagnosis for plantar heel pain, enhances the clinical relevance and enables a nuanced understanding of biomarker variations across conditions.

### Psychosocial Factors in Myofascial Pain

In addition to examining biological factors contributing to myofascial pain, the study will also explore how psychosocial factors may enhance the accuracy of an imaging biosignature. Psychosocial factors have been shown to influence pain perception and treatment response, further complicating diagnosis. By expanding the definition of a “biosignature” to include psychological variables such as depression, anxiety, and fear of movement, this study aligns with emerging trends in biomarker research, which recognize the interplay of biological and psychological factors in pain chronicity [76].

### Limitations

A limitation of this study is the absence of a universally accepted definition of a trigger point across body regions and between professions. In this study, the definition of a trigger point is based on criteria commonly cited in the field of physical therapy [12,13]. Yet other clinicians and researchers examining myofascial pain may select other definitions. As evidence on biosignatures of myofascial pain continues to emerge, these findings will help refine and standardize the definition of trigger points across diagnoses and professions.

### Implications for Future Research and Clinical Practice

The anticipated findings will lay the groundwork for future large-scale investigations, including clinical trials focused on nonpharmacological and nonsurgical interventions for plantar heel pain, such as dry needling. By establishing a robust biosignature for myofascial pain, this research has the potential to refine diagnostic criteria, improve treatment selection, and contribute to a more personalized approach to pain management.

### Conclusions

This protocol provides novel mechanistic insights into myofascial pain through advanced imaging, offering a biopsychosocial framework for improved diagnosis and treatment of plantar heel pain and related conditions. We anticipate that combining imaging and psychosocial measures will improve the diagnostic accuracy of the biosignature and provide a more comprehensive understanding of myofascial pain.

## Appendix

Multimedia Appendix 1 Peer review report by ZAT1 SH (14) - National Center for Complementary and Integrative Health (NCCIH) Special Emphasis Panel HEAL Initiative: Toward Developing Quantitative Imaging and Other Relevant Biomarkers of Myofascial Tissues for Clinical Pain Management (National Institutes of Health, USA).

## Abbreviations

HIPAA: Health Insurance Portability and Accountability Act
IDEAL: Iterative Decomposition of water and fat with Echo Asymmetry and Least-squares estimation–Quantitative Imaging
IVIM: intravoxel incoherent motion
MRI: magnetic resonance imaging
NRS: numeric rating scale
ROC AUC: area under the receiver operating characteristic curve
ROI: regions of interest
SWE: shear wave elastography

## Data Availability

In compliance with the findability, accessibility, interoperability, and reusability principles, data will be deposited in the University of Iowa’s open access institutional repository, Iowa Research Online. This repository is maintained by the University of Iowa Libraries to preserve and share scholarly work. Individual participant data will be shared upon publication. Primary outcome data and all information necessary to validate the research findings of this study will be preserved and made available to support reproducibility and facilitate further research that builds upon these results.
