# Supplementary material for: Quantitative Understanding of Advanced Novel Imaging Techniques for Fasciitis and Biosignature Yield (Quantify): Protocol for a Cross-Sectional Diagnostic Study
Source: JMIR Res Protoc. 2026 Feb 11;15:e87613. doi: 10.2196/87613 (PMC12936652; doi:10.2196/87613)
Supplement: Multimedia Appendix 1 [file resprot_v15i1e87613_app1.pdf]

SUMMARY STATEMENT

(Privileged Communication)

PROGRAM CONTACT:
Dr. Alexander Tuttle
301-496-2583
alex.tuttle@nih.gov

Release Date: 03/03/2024
Revised Date:

Application Number: 1R61AT012275-01A1
Formerly: 1R61AT012275-01

Principal Investigators (Listed Alphabetically):

CHIMENTI, RUTH LOUISE PORTER (Contact)
HOLMES, JAMES H
SLUKA, KATHLEEN A

Applicant Organization: UNIVERSITY OF IOWA

Review Group: ZAT1 SH (14)
National Center for Complementary and Integrative Health Special Emphasis Panel
HEAL Initiative: Toward Developing Quantitative Imaging and Other Relevant Biomarkers of Myofascial Tissues for Clinical Pain Management (R61/R33, Clinical Trial Required)

Meeting Date: 02/16/2024
Council: MAY 2024
Requested Start: 07/01/2024

Opportunity Number: RFA-AT-24-003
PCC: TUTTLEA
Dual PCC: 3 B
Dual IC(s): AR, HD, NS

Project Title: Development of diagnostic and prognostic ultrasound imaging biomarkers for plantar heel pain
SRG Action: Impact Score:36
Next Steps: Visit https://grants.nih.gov/grants/next_steps.htm
Human Subjects: 30-Human subjects involved - Certified, no SRG concerns
Animal Subjects: 10-No live vertebrate animals involved for competing appl.
Gender: 1A-Both genders, scientifically acceptable
Minority: 1U-Minorities and non-minorities, scientifically unacceptable
Age: 3A-No children included, scientifically acceptable

Project Year	Direct Costs Requested	Estimated Total Cost
1	480,701	736,945
2	481,486	738,149
3	667,190	1,022,845
4	674,896	1,034,659
5	637,386	977,154
TOTAL	2,941,659	4,509,752

1R61AT012275-01A1 Chimenti, Ruth

INCLUSION OF MINORITIES PLAN UNACCEPTABLE

RESUME AND SUMMARY OF DISCUSSION: This R61/R33 resubmission application titled "Development of diagnostic and prognostic ultrasound imaging biomarkers for plantar heel pain" is submitted in response to RFA-AT-24-003 "HEAL Initiative: Toward Developing Quantitative Imaging and Other Relevant Biomarkers of Myofascial Tissues for Clinical Pain Management (R61/R33, Clinical Trial Required)" by the University of Iowa with Drs. Ruth Chimenti, James Holmes, and Kathleen Sluka as the principal investigators. This application proposes to develop imaging biomarkers for myofascial tissues associated with planter fasciitis using methods including ultrasound shear wave elastography (SWE), strain imaging, and magnetic resonance imaging (MRI) in the R61 phase, and to determine predictive and responsive imaging biomarkers in a clinical trial comparing active and sham dry needling treatments in patients with plantar fasciitis. This is a strong application that aims to address a common musculoskeletal pathology that is often recalcitrant to treatment approaches. If successful, the quantitative biomarkers would provide a more objective assessment of myofascial pain compared to current available clinical examination and could enable better treatment targeting. While the proposed imaging techniques individually are not novel, the combined use of MRI and ultrasound imaging is viewed as a unique approach. The development of imaging biomarkers that would assist with the diagnosis and treatment prescription of plantar fasciitis is also deemed innovative. However, it is noted that it is unclear whether all patients with plantar fasciitis demonstrate myofascial pain and related trigger points. It is also unclear whether the techniques applied in this study can be easily adapted to other myofascial pain conditions, raising concerns about the generalizability of the study's findings. The investigative team led by three multiple principal investigators is outstanding with multidisciplinary expertise in physical medicine and rehabilitation, radiology, statistics, and machine learning. The environment is excellent with well-established clinical and research facilities, state-of-the-art equipment, and other resources available to successfully carry out the proposed research. Overall, the study design is rigorous with details focusing on the reproducibility of findings. The inclusion of an insertional Achilles tendinopathy that would help in differential diagnosis is deemed a strength. Notably, evaluating imaging biomarkers at multiple time points is considered a strength as it would facilitate an adequate assessment of the response to treatment. Further, the inclusion of psychological measures in conjunction with biological markers as outcomes in the clinical trial of the R33 phase is also deemed a strength. The inclusion/exclusion criteria are considered well-delineated, and sex as a biological variable would be addressed by recruiting men and women equally. Moreover, the statistical analysis plan for both phases is thoroughly explained. While the resubmission is largely viewed as responsive to previous critiques, resulting in an overall improved application, several additional weaknesses in the proposed approach are also noted. For example, it is unclear whether the investigators have access to a large enough patient pool for the clinical study in the R33 phase. The sham dry needling procedure may not be an adequate placebo control and could potentially bias the placebo group as concerns are raised about the subject blinding for a sham non-penetrative dry needling procedure if the actual dry needling procedure is potentially painful and requires the twitch response. Further, there is limited evidence supporting that the changes of ultrasound and particularly MRI biomarkers by dry needling reflect true pathologic changes and potential treatment responses. Additionally, the MRI techniques proposed would likely not adequately capture changes in muscle or fascia. As MRI would be performed on both sides with the symptomatic side as control, it is unclear how the investigators would handle patients that may have bilateral and potentially subclinical pathology. Notably, the analysis plan for the SWE and MRI could have been better described. While the milestones are in general appropriate, there is a concern about the threshold for the area under the receiver operating characteristics curve, and a more rigorous definition of the quantitative transition criteria is needed. In conclusion, this is an

innovative resubmission application from a strong investigative team with access to an exceptional research environment aimed at developing promising imaging biomarkers for myofascial pain in plantar fasciitis. While generally responsive to the previous review, some remaining issues centering around aspects related to the study design, particularly related to the MRI techniques, and the transition criteria slightly diminish the enthusiasm for this resubmitted application.

DESCRIPTION (provided by applicant): Myofascial pain remains an underdiagnosed contributor to a range of musculoskeletal pain conditions. The lack of validated biomarkers limits the ability to objectively detect myofascial pain, probe underlying pain mechanisms, and guide targeted treatments. This proposal will address this gap by quantifying the biochemical and biomechanical properties of myofascial pain using advanced, quantitative imaging techniques. As a model of myofascial pain, we have chosen plantar fasciitis, which affects 1 out of every 10 adults. Our long-term goal is to enhance musculoskeletal pain management by creating better tools for detecting abnormal myofascial tissue that enable more individualized treatment. The objective of the R61 phase is to use novel imaging techniques to develop a diagnostic biomarker to objectively and accurately determine the location and severity of abnormal myofascial tissue. Our approach will use a cross-sectional study design with 3 groups: plantar fasciitis (n=40), Achilles tendinopathy (n=20), and pain-free controls (n=20) to test Specific Aim 1: Develop diagnostic imaging biomarkers of myofascial tissue to differentiate individuals with plantar fasciitis from other foot pain without a myofascial component (Achilles tendinopathy) and from matched pain-free controls. The objective of the R33 phase is to use novel imaging techniques to develop a predictive biomarker to identify individuals most likely to respond to DN, and a response biomarker to guide dosing or continued use of DN for myofascial pain for individuals with plantar foot pain. Our approach will use a parallel-group, double-blinded randomized controlled trial (RCT) design with imaging biomarkers measured before, during (1 month), and after treatment (short-term: 3 months (Primary Endpoint), longer-term: 6 months). Participants will be randomized to one of two groups: 1) DN + standard care, or 2) Sham DN + standard care to test Specific Aim 2: Determine predictive and response imaging biomarkers in an RCT. For both R61 and R33 phases, Exploratory Aim 3: Will develop composite biosignatures that combine multiple imaging biomarkers, developed in isolation in Aims 1 or 2, with or without psychosocial factors, to enhance the diagnostic, predictive, or response capability for myofascial pain. Transition criteria from R61 to R33 that must be met by at least month 20 within the R61 phase: 1) Adequate recruitment with >90% of participants in each group enrolled; 2) Adequate representation with neither sex exceeding 60% of the sample; 3) Minimal missing data (<5%) for collected outcomes; 4) At least one diagnostic imaging biomarker with an area under the receiver operating characteristics curve (ROC AUC) > 0.7; 5) Complete all planning activities required to implement the R33 phase RCT.

PUBLIC HEALTH RELEVANCE: This research addresses the pressing issue of underdiagnosed myofascial pain, which plays a significant role in various musculoskeletal pain conditions, including plantar fasciitis. By developing novel imaging biomarkers, this study aims to provide objective means of detecting myofascial pain, understanding its underlying mechanisms, and guiding personalized treatments. Given the high prevalence of plantar fasciitis and its long-lasting impact, this research has the potential to improve the diagnosis and treatment of not only this condition but also other lower extremity musculoskeletal disorders, ultimately enhancing public health by reducing illness and disability associated with chronic pain.

CRITIQUE 1

Significance: 2
Investigator(s): 1

Innovation: 3
Approach: 3
Environment: 1

Overall Impact:
This is a resubmission of a project that seeks to develop ultrasound (US) shear wave elastography (SWE), strain imaging and magnetic resonance imaging (MRI) T1-rho imaging-based biomarkers for plantar fasciitis in the R61 phase, and then evaluate these biomarkers in a clinical trial of dry needling in the R33 phase. The previous review identified a number of strengths of the application, including the novel use of quantitative imaging methods for plantar fasciitis, a well-designed study, a well-qualified team, and an excellent environment. Weaknesses were identified in the significance of myofascial trigger points in plantar fasciitis, a potentially painful intervention limiting feasibility of the R33 phase, and an incremental advance in technology. In the resubmission application, the investigators have addressed a number of these concerns. They have added MRI imaging metrics (as well as preliminary data supporting feasibility). The investigative team has been strengthened even further by adding Drs. Sluka (translational pain research), Holmes (imaging) and Smith (data science). The modifications have addressed many of the major weaknesses, although a few minor weaknesses remain.

1. Significance:
Strengths
- The investigators have made a compelling argument that myofascial dysfunction may be under appreciated in plantar fasciitis.
- The selection of a specific anatomical region with a high prevalence of soft tissue dysfunction is compelling and has the potential to generate valuable knowledge that might be generalizable to other anatomical sites.
- The combination of US SWE, strain, and multiparametric MRI imaging is comprehensive and would yield valuable data.
- There is a gap in knowledge about which individuals with plantar fasciitis are likely to benefit from dry needling, and the proposed biomarkers can enable better targeting the treatment.

Weaknesses
- For the R61 phase, the investigators argued that the biomarkers may be used to guide who would be good candidates for dry needling. However, the design of the R33 study does not address this issue. It is unclear how the biomarkers would be utilized to determine suitable candidates for treatment, i.e., it is unclear how baseline biomarkers would be used to identify responders from non-responders. The study does not seem to be powered for this outcome.
- The presence of myofascial trigger points (MTrPs) in the calf muscles associated with plantar fasciitis would indicate alterations in the myofascial chain. Focusing the imaging only to the MTrPs may be a missed opportunity to uncover the linkage between the two.

2. Investigator(s):
Strengths
- This is an outstanding team of investigators with complementary expertise. The team includes three multiple principal investigators: Dr. Sluka (expert on translational pain research), Dr. Chimenti (clinical scientist), and Dr. Holmes (expert on imaging).
- The team also includes experts on clinical trials and biostatisticians who provide complementary expertise and have contributed to a well-thought-out clinical trial plan.
- The addition of Dr. Donnelly (co-editor of the Travell and Simons' manual) to advise on the relevance of MTrPs in plantar fasciitis is a strength.

Weaknesses
- No major weaknesses noted.

3. Innovation:
Strengths
- The use of multiple imaging modalities to study biomechanical and biochemical aspects of the myofascial tissues is innovative and would lead to new data.

Weaknesses
- The proposed work utilizes conventional imaging-based biomarkers that have been previously reported and was no real innovation on the imaging side.
- While the proposed study would generate a lot of interesting data, there was no real innovation in the methods of data analysis that might lead to new knowledge by integrating complementary data.

4. Approach:
Strengths
- It is a rigorous study design for the R61 and R33 phases.
- An important strength of the study design is the inclusion of an insertional Achilles tendinopathy as a comparator that would help in differential diagnosis with the biomarkers.
- The study focuses on a specific anatomical region, with attention to detail on reproducibility of findings.
- There are rigorous inclusion and exclusion criteria.
- The addition of the strain imaging of the Achilles tendon would be useful to differentiate between the three groups.
- There is careful attention to detail in proposed planning activities for the R61 to R33 transition.

Weaknesses
- It is unclear whether the investigators have access to a large enough patient pool for the interventional study in the R33 phase. The application mentions ~20 subjects/year undergo dry needling for plantar fasciitis at their institution.

The sham dry needling procedure may not be an adequate placebo control. It is unclear how the investigators can keep the subject blinded for a sham non-penetrative dry needling procedure if the actual dry needling procedure is potentially painful and requires the twitch response. This has the potential to bias the placebo group. The lack of a suitable sham dry needling procedure is a problem for the field in general, and the application does not adequately address it.

- It is unclear how the shear wave images would be analyzed to generate a biomarker. The provided example shows the increased shear speed in the fascia between the gastrocnemius and soleus muscles. It is unclear how this would be related to the MTrPs.
- As pointed out in a previous review, the investigators are not considering biomarkers extracted from B-mode imaging, which may be a missed opportunity.

5. Environment:
Strengths
- The environment is outstanding, and the investigators have all the resources available to successfully conduct this proposed work.

Weaknesses
- No major weaknesses noted.

Study Timeline:
Strengths
- There is a very detailed timeline, with carefully thought-out details for the transition planning.

Weaknesses
- No major weaknesses noted.

Protections for Human Subjects:
Acceptable Risks and/or Adequate Protections
Data and Safety Monitoring Plan Applicable for Clinical Trials Only:
Acceptable
- The data safety monitoring plan (DSMP) would be fleshed out for the R33 phase.

Inclusion of Women, Minorities, and Individuals Across the Lifespan:
- Sex/Gender: Distribution justified scientifically
- Race/Ethnicity: Distribution justified scientifically
- For NIH-Defined Phase III trials, plans for valid design and analysis: Not applicable
- Inclusion/Exclusion Based on Age: Distribution justified scientifically
- The investigator has paid attention to sex as a biological variable. Children are excluded since plantar fasciitis is rare in children.

Vertebrate Animals:
Not Applicable (no vertebrate animals)

Biohazards:
Not Applicable (No Biohazards)

Resubmissions:
- The resubmission has addressed many of the concerns raised in the prior review.

Milestones:
- The transition criteria include a quantitative metric of area under the receiver operating characteristic curve (ROC AUC) exceeding 0.7 for at least one biomarker. The concern is that just specifying an AUC threshold without any constraints on sensitivity or specificity could lead to the selection of a biomarker that might not be useful in the R33 phase, or useful clinically. A more rigorous definition of the quantitative transition criteria is needed.

Applications from Foreign Organizations:
Not Applicable (No Foreign Organizations)

Select Agent Research:
Not Applicable (No Select Agents)

Resource Sharing Plans:
Not Applicable (No Relevant Resources)

Authentication of Key Biological and/or Chemical Resources:
Not Applicable (No Relevant Resources)

Budget and Period of Support:
Recommend as Requested

CRITIQUE 2

Significance: 4

Investigator(s): 2
Innovation: 6
Approach: 6
Environment: 1

Overall Impact:
R61 phase: The strengths of the R61 phase approach are in its consideration of multiple different modalities [magnetic resonance imaging (MRI), ultrasound] for tissue evaluation of the lower leg and foot. Ultrasound and MRI are overseen by experts in the field and the team has extensive experience applying ultrasound for the evaluation of both plantar fasciitis and Achilles tendinosis/tear. The proposed MRI techniques, however, are not deemed to be sensitive to microstructural changes of plantar fasciitis or tendinosis.
R33 phase: The strengths include a randomized controlled trial (RCT) that includes a sham group, but also enables participants to undergo standard care to alleviate their pain. Additionally, the study provides for longitudinal evaluation of patients prior to, during, and following treatment conclusion. No additional weaknesses beyond those described for the R61 phase and applied to the R33 phase are considered here.
Summary: Plantar fasciitis is a very common musculoskeletal pathology that leads to pain and there is evidence to support that myofascial dysfunction is integral in its development. In its first (R61) phase, the study aims to determine which quantitative imaging biomarkers, when also correlating with psychosocial factors, would reliably depict macro- and microstructural changes of muscle and fascial tissue that reflect pain-inducing trigger points in plantar fasciitis. In its second (R33) phase, it aims to determine predictive and responsive imaging biomarkers in an RCT of patients undergoing 'real' versus 'sham' dry needling. This study, if successful, may develop more objective criteria to triage patients into the appropriate treatment category and provide more objective data to determine the efficacy of dry needling. There is overall a low level of enthusiasm for this study project, however, as the MRI techniques outlined in the application are not considered to be effective in detecting myofascial changes. As such, their successful application in the study of plantar fasciitis and generalizability to other myofascial-related pain conditions are predicted to be poor.

1. Significance:
Strengths
- Plantar fasciitis is very common and causes pain that sometimes cannot be alleviated with non-invasive treatment.
- Quantitative biomarkers inherently provide more objective assessment of myofascial pain compared to clinical examination alone, which mostly involves manual palpation to reproduce symptoms and identify trigger points.

Weaknesses
- There is concern about the generalizability of the study's findings,i.e., it is unclear whether the techniques applied in this study can be easily adapted to study other myofascial pain conditions, as the investigators suggest.

2. Investigator(s):
Strengths
- The principal investigator leads a multidisciplinary team comprising specialists in physical medicine and rehabilitation, radiology, and statistics, including machine learning. Dr. Chimenti (principal investigator) and Dr. Sluka (multiple principal investigator) have extensive collaborative experience, as well as specific experience in studying plantar fasciitis, Achilles pathology, and myofascial pain and applying ultrasound for foot and ankle clinical research. The team also includes Dr. Holmes (multiple principal investigator), who has extensive experience in

advanced quantitative MRI techniques, and Dr. Richards (co-investigator) with ultrasound expertise. In summary, the structure of the study team is logical with experts in all study domains.

Weaknesses

- The team also includes Dr. Holmes (multiple principal investigator) with no previous collaborative publishing experience with Drs. Chimenti or Sluka.

3. Innovation:

Strengths

- The assessments are broad and include both multimodality imaging assessments as well as measures of psychosocial dysfunction.
- Imaging assessment response to dry needling for the condition of plantar fasciitis has not been well-studied. The consideration of two different imaging modalities is commendable.

Weaknesses

- None of the proposed imaging techniques are novel, in of themselves. For example, shear wave elastography (SWE) has been studied for many conditions and MR T2 mapping/T1 rho techniques have been around for many decades.

4. Approach:

Strengths

- For the R61 phase, the investigators emphasize relatively equal recruitment of men and women (to evaluate influence of sex as a biological variable, as women are twice as likely to have plantar fasciitis compared to men) and include comparison of plantar fasciitis, insertional Achilles tendinopathy, and asymptomatic cohorts. For the R33 phase, this is a double-blinded placebo-controlled study.
- For ultrasound, displacement measurements to quantify average shear strain with plantar fascia would be repeated three times to ensure accuracy.
- Clear transition and inclusion/exclusion criteria are delineated.
- For the R33 clinical trial design, the investigators would evaluate imaging biomarkers at multiple time points (before, during, and after treatment) thus facilitating an adequate assessment of the response to treatment.

Weaknesses

- Achilles tendinosis and plantar fasciitis present differently, the former with ankle pain and the latter more frequently with foot pain. As such, the question arises whether imaging biomarkers are truly needed to differentiate the two and whether Achilles tendinosis is an appropriate comparison group. Perhaps a more suitable group would be patients with acquired flatfoot deformity.
- There is limited evidence provided by the investigators to support that dry needling changes ultrasound and particularly MRI biomarkers and reflects true pathologic changes and potential treatment responses.
- MRI: The techniques applied in this application would likely not adequately capture changes in muscle or fascia, if present. Firstly, zero echo-time (ZTE) imaging is a technique used to visualize the short T2-species of cortical bone to create CT-like images and its ability to reliably depict the plantar fascia is uncertain. Its use here is not substantiated as it is not a quantitative technique and measurement of plantar fascia thickness and volume can be easily obtained by more conventional proton density imaging. ZTE is unlikely to provide reliable assessment of calcifications, as ZTE is not very sensitive to calcification, and calcification (if at all present) would be at the microscopic level.
- Protocol parameters for T2 mapping and T1 rho are not provided. Conventional T2 mapping techniques are likely inadequate to capture changes in the rapidly decaying short T2 species of

the Achilles tendon and the plantar fascia itself or to detect fibrosis/trigger points in muscle tissue; ultrashort echo time (UTE) sequences, instead, would be more sensitive to such changes.
- The rationale for measuring fatty infiltration is not provided. Additionally, the rationale for using a time-intensive, 2-point Dixon approach as compared to 6-point (IDEAL) is not obvious.
- MRI would be performed on both sides, with the symptomatic side considered a ‘control’. However, it is unclear how the study investigators would handle patients that may have bilateral and potentially subclinical pathology.
- For MRI analysis: it is unclear how the investigators would handle situations in which no ‘T2 hyperintensities’ are identified as the study calls for placing regions of interest (ROIs) around such regions to direct ROIs on the T1rho sequence.

5. Environment:
Strengths
- The institution and its specialized clinical and research facilities are well-established and have state-of-the-art equipment to carry out the proposed research.

Weaknesses
- No major weaknesses noted.

Study Timeline:
Strengths
- The study timeline is concisely laid out and there is time allotted for planning for the second phase of study.

Weaknesses
- No major weaknesses noted.

Protections for Human Subjects:
Acceptable Risks and/or Adequate Protections
Data and Safety Monitoring Plan Applicable for Clinical Trials Only:
Acceptable

Inclusion of Women, Minorities, and Individuals Across the Lifespan:
- Sex/Gender: Distribution justified scientifically
- Race/Ethnicity: Distribution justified scientifically
- For NIH-Defined Phase III trials, plans for valid design and analysis: Not applicable
- Inclusion/Exclusion Based on Age: Distribution justified scientifically
- Adequate

Vertebrate Animals:
Not Applicable (no vertebrate animals)

Biohazards:
Not Applicable (No Biohazards)

Resubmissions:
- The summary sheet nicely summarizes changes made within the resubmission.

Milestones:
- Yes, the milestones are appropriate.

Resource Sharing Plans:
Not Applicable (No Relevant Resources)

Budget and Period of Support:
Recommend as Requested

CRITIQUE 3

Significance: 3
Investigator(s): 3
Innovation: 4
Approach: 4
Environment: 1

Overall Impact:
The investigators propose a two-stage investigation using the R61/R33 mechanism. The objective of the R61 phase is to identify imaging biomarkers that identify myofascial tissue dysfunction in patients with plantar fasciitis. Both magnetic resonance imaging (MRI) and ultrasound imaging techniques would be used to compare patients with plantar fasciitis to a group with Achilles tendinopathy and a healthy control group. The objective of the R33 phase is to perform a randomized controlled trial (RCT) comparing active dry needling to sham dry needling in patients with plantar fasciitis. The outcome measures would include imaging biomarkers and patient-reported outcome measures including psychological measures. There are potential scaffolding concerns that the R61 phase fails to identify one or more definitive imaging biomarkers. It is also not clear how prevalent myofascial dysfunction is in patients with plantar fasciitis, thus representing a potential weakness.

1. Significance:
Strengths
- Plantar fasciitis is a common musculoskeletal pathology that is often recalcitrant to common treatment approaches.
- Dry needling is an emerging therapeutic intervention for many musculoskeletal soft tissue pathologies; however, the evidence base of its efficacy is not large.

Weaknesses
- It is unclear if all patients with plantar fasciitis demonstrate myofascial pain and related trigger points. If there are different subgroupings of plantar fasciitis presentation with and without trigger points (or other myofascial dysfunction), there may be concerns about the design of the two studies (particularly in regard to statistical power).

2. Investigator(s):
Strengths
- The research team is led by three principal investigators, each with unique expertise. There is also a strong team of co-investigators.
- Dr. Chimenti is an assistant professor and physical therapist who has a good record of lower extremity injury research and working on extramurally funded research.
- Dr. Sluka is an established investigator with a strong record of leading large and funded research projects related to musculoskeletal pain. She is a physical therapist with extensive research experience.
- Dr. Holmes is an assistant professor and imaging researcher who has a good record of external funding supporting applied imaging research.

- Dr. Chimenti and Dr. Sluka have a record of previous collaboration.

Weaknesses

- Dr. Holmes has limited collaboration experience with Drs. Chimenti and Sluka. This is viewed as a minor concern that is balanced by Dr. Sluka's vast experience in leading large collaborative research projects.

3. Innovation:

Strengths

- The objective to develop MRI and ultrasound imaging markers of myofascial dysfunction in plantar fasciitis patients is novel and innovative.
- The development of one or more imaging biomarkers that would assist with the diagnosis and treatment prescription of plantar fasciitis is innovative.
- The combined use of MRI and ultrasound imaging represents a unique approach in an effort to identify useful imaging biomarkers.
- The use of dry needling as the primary intervention to be assessed is appropriate as this is an emerging conservative treatment approach that has a limited evidence base.
- The attempt to identify diagnostic, predictive, and response biomarkers is ambitious and innovative.

Weaknesses

- This reviewer has some reservations that all patients with plantar fasciitis would demonstrate signs of myofascial dysfunction that can be assessed via imaging. It is not clear whether plantar fasciitis is always a myofascial issue, as opposed to a fascial-only problem.

4. Approach:

Strengths

- The R61's comparison of three groups (plantar fasciitis, Achilles tendinopathy, and healthy controls) is commendable because of the inclusion of the Achilles group that is projected to not have myofascial dysfunction.
- The proposed imaging outcome measures using both MRI and ultrasound measures should provide a thorough assessment of myofascial tissue quality and quantity.
- The standard of care intervention that both groups would receive in the RCT contains appropriate exercises and development of develop patient-specific physical activity goals by the treating physical therapist.
- The inclusion of psychological measures in conjunction with biological markers as outcomes in the RCT is a strength.
- The statistical analysis plan for both sections is thoroughly explained.

Weaknesses

- There is the potential for a scaffolding problem in which appropriate outcomes are not found in the R61 study.
- The threshold of area under the receiver operating characteristics curve (AUC)>0.7 in the R61 analysis raises concern because the proportion of false positives and/or false negatives can still be fairly high with that AUC threshold. There are concerns if only a single imaging biomarker were identified, and the associated AUC was only slightly above 0.7.
- While the Optimal Screening for Prediction of Referral and Outcome Yellow Flags (OSPRO-YF) survey instrument contains some items from the Tampa Scale of Kinesiophobia (TSK), it is unclear if the TSK should be administered in its entirety as outcome measure in the R33 phase.

5. Environment:

Strengths

- Adequate facilities are described to execute all aspects of the proposed research.

Weaknesses

- No major weaknesses noted.

Study Timeline:

Strengths

- The timeline appears to be appropriate.

Weaknesses

- No major weaknesses noted.

Protections for Human Subjects:

Acceptable Risks and/or Adequate Protections

Data and Safety Monitoring Plan Applicable for Clinical Trials Only:

Acceptable

Inclusion of Women, Minorities, and Individuals Across the Lifespan:

- Sex/Gender: Distribution justified scientifically
- Race/Ethnicity: Distribution not justified scientifically
- For NIH-Defined Phase III trials, plans for valid design and analysis: Not applicable
- Inclusion/Exclusion Based on Age: Distribution justified scientifically
- Sex distribution limits acknowledge that plantar fasciitis is more common in females.
- Race/ethnicity distributions are estimated off of regional population at the site.

Vertebrate Animals:

Not Applicable (no vertebrate animals)

Biohazards:

Not Applicable (No Biohazards)

Resubmissions:

- The responses to previous concerns are clear.

Milestones:

- The milestones appear to be appropriate.
- As previously mentioned, the threshold of AUC>0.7 in the R61 analysis raises concern because the proportion of false positives and/or false negatives can still be fairly high with that AUC threshold. There would be concerns if only a single imaging biomarker is identified and the associated AUC was only slightly above 0.7.

Budget and Period of Support:

Recommend as Requested

CRITIQUE 4

Overall Impact:

In the R61 phase, the applicants propose evaluating 10 imaging measures [ultrasound and magnetic resonance imaging (MRI)-based] with two statistics to identify the top five most promising biomarkers for evaluating abnormal myofascial tissue associated with plantar fasciitis. The significance of the project is high, as plantar fasciitis is associated with myofascial trigger points in the foot and calf in many individuals. The diagnosis would likely be applicable to other conditions with myofascial trigger

points. Similarly, the investigative team is strong, as are the scientific environments at the University of Iowa and Rochester Institute of Technology. The innovation of the R61 phase is moderately high, as while most of the ultrasound and MRI technologies have been used to evaluate active myofascial trigger points, they have not been used in combination, nor has shear strain (ultrasound) nor T1rho MRI been used to evaluate the trigger points. The approach section is also strong, although a few concerns were noted including the non-testable/interesting hypothesis for Aim1; confusion of the number and criteria for choosing which quantitative metrics would be chosen for the R33 phase; a relatively low 0.7 area under the receiver operating characteristics (ROC) curve (AUC) needed as the milestone criterion; questions as to whether the goal is to distinguish between active and latent trigger points; and the use of exercise logs/patient compliance. Overall, these concerns in the approach were found to be minor to moderate and likely related to the fixed page limit of the application. The applicants were also very responsive to prior critiques. The transition criteria for R61 to R33 were quantifiable and clear, although only one criterion (>0.7 ROC) was directly related to the quantitative metrics developed in the R61 phase. In the R33 phase, the applicants propose conducting a parallel group, double-blind randomized controlled trial (RCT) where dry needling with exercise and stretching is compared to sham dry needling (still with exercise and stretching). The chosen intervention is clinically used, and the applicants propose four time points for evaluation:baseline, mid-treatment, 3 months, and 6 months. The 6-month follow-up is very compelling. The investigators also include an exploratory third aim that uses machine learning to combine the evaluated biomarkers in an attempt to evaluate predictive and response biomarkers for myofascial trigger pain. Summary: Enthusiasm for the application remains very high, as only a few minor-moderate concerns were identified in the approach, keeping the potential impact of the project high.

CRITIQUE 5

Overall Impact:
The research team plans to determine the location and severity of abnormal myofascial tissue using a cross-sectional study design with three groups. In the R33 phase, the team plans to use imaging techniques to develop a predictive biomarker to identify individuals most likely to respond to dry needling (DN), and a response biomarker to guide dosing or continued use of DN for myofascial pain for individuals with plantar foot pain. Furthermore, in the R33 phase, the team plans to use a parallel-group, double-blinded randomized controlled trial (RCT) design with imaging biomarkers measured before, during (1 month), and after treatment (short-term: 3 months (Primary Endpoint), longer-term: 6 months). Participants would be randomized to one of two groups: 1) DN standard care, or 2) Sham DN standard care to test. Dr. Smith with rich experience in imaging and clinical trial is identified as a biostatistician with 10% effort for the duration of the study. The analysis plans for both R61 and R33 phases are delineated, and the explanations for the justification of sample size are provided. For the R61 phase, the diagnostic performances of each of the 20 candidate biomarkers would be assessed using the area under the receiver operating characteristics curve (ROC AUC) in differentiating active muscle trigger points (A-MTrPs) in plantar fasciitis from healthy tissue, employing 5-fold cross-validation approach. Up to 5 top performers, with pre-defined criteria, would be selected for the R33 clinical trial. Additional analytical strategy is described. The R61 to R33 transition criteria are well defined. For the R33 phase, intention-to-treat analysis using linear mixed model analysis to assess the significance of the imaging biomarkers in predicting the change in pain intensity is provided. No weaknesses are noted.

THE FOLLOWING SECTIONS WERE PREPARED BY THE SCIENTIFIC REVIEW OFFICER TO SUMMARIZE THE OUTCOME OF DISCUSSIONS OF THE REVIEW COMMITTEE, OR REVIEWERS' WRITTEN CRITIQUES, ON THE FOLLOWING ISSUES:

PROTECTION OF HUMAN SUBJECTS: ACCEPTABLE

INCLUSION OF WOMEN PLAN: ACCEPTABLE

INCLUSION OF MINORITIES PLAN: UNACCEPTABLE
The race/ethnicity distributions are estimated off of regional population at the site.

INCLUSION ACROSS THE LIFESPAN: ACCEPTABLE

COMMITTEE BUDGET RECOMMENDATIONS: The budget was recommended as requested.

Footnotes for 1R61AT012275-01A1; PI Name: Chimenti, Ruth Louise Porter

NIH has modified its policy regarding the receipt of resubmissions (amended applications).See Guide Notice NOT-OD-18-197 at https://grants.nih.gov/grants/guide/notice-files/NOT-OD-18-197.html. The impact/priority score is calculated after discussion of an application by averaging the overall scores (1-9) given by all voting reviewers on the committee and multiplying by 10. The criterion scores are submitted prior to the meeting by the individual reviewers assigned to an application, and are not discussed specifically at the review meeting or calculated into the overall impact score. Some applications also receive a percentile ranking. For details on the review process, see http://grants.nih.gov/grants/peer_review_process.htm#scoring.

MEETING ROSTER

National Center for Complementary and Integrative Health Special Emphasis Panel
NATIONAL CENTER FOR COMPLEMENTARY & INTEGRATIVE HEALTH
HEAL Initiative: Toward Developing Quantitative Imaging and Other Relevant Biomarkers of Myofascial Tissues for Clinical Pain Management (R61/R33, Clinical Trial Required)
ZAT1 SH (14)
02/16/2024

Notice of NIH Policy to All Applicants: Meeting rosters are provided for information purposes only. Applicant investigators and institutional officials must not communicate directly with study section members about an application before or after the review. Failure to observe this policy will create a serious breach of integrity in the peer review process, and may lead to actions outlined in NOT-OD-22-044 at https://grants.nih.gov/grants/guide/notice-files/NOT-OD-22-044.html, including removal of the application from immediate review.

CHAIRPERSON(S)

APKARIAN, A. VANIA, PHD
PROFESSOR AND DIRECTOR
DEPARTMENTS OF NEUROSCIENCE, ANESTHESIOLOGY AND PHYSICAL MEDICINE AND REHABILITATION
FEINBERG SCHOOL OF MEDICINE
NORTHWESTERN UNIVERSITY
CHICAGO, IL 60611

EHMAN, RICHARD L, MD
PROFESSOR
DEPARTMENT OF RADIOLOGY
MAYO CLINIC
ROCHESTER, MN 55905

MEMBERS

AJIT, SEENA, PHD
PROFESSOR
DEPARTMENT OF PHARMACOLOGY AND PHYSIOLOGY
COLLEGE OF MEDICINE
DREXEL UNIVERSITY
PHILADELPHIA, PA 19102

BAE, SEJONG, PHD
PROFESSOR
DEPARTMENT OF MEDICINE
DIVISION OF PREVENTIVE MEDICINE
HEERSINK SCHOOL OF MEDICINE
UNIVERSITY OF ALABAMA AT BIRMINGHAM
BIRMINGHAM, AL 35205

BRUMMETT, CHAD M, MD
PROFESSOR AND SENIOR ASSOCIATE CHAIR
DEPARTMENT OF ANESTHESIOLOGY
UNIVERSITY OF MICHIGAN MEDICAL SCHOOL
ANN ARBOR, MI 48108

CEVIDANES, LUCIA H, PHD, DDS
ENDOWED PROFESSOR
DEPARTMENT OF ORTHODONTICS AND PEDIATRIC DENTISTRY
SCHOOL OF DENTISTRY
UNIVERSITY OF MICHIGAN
ANN ARBOR, MI 48109

CHOLEWICKI, JACEK, PHD
PROFESSOR
DEPARTMENT OF OSTEOPATHIC MANIPULATIVE MEDICINE
CENTER FOR NEUROMUSCULOSKELETAL CLINICAL RESEARCH
COLLEGE OF OSTEOPATHIC MEDICINE
MICHIGAN STATE UNIVERSITY
EAST LANSING, MI 48824

CORTES CORREALES, DANIEL H, PHD
ASSOCIATE PROFESSOR
DEPARTMENT OF MECHANICAL ENGINEERING
MATERIALS RESEARCH INSTITUTE
THE PENNSYLVANIA STATE UNIVERSITY
UNIVERSITY PARK, PA 16802

DIXON, J. BRANDON, PHD
PROFESSOR AND ASSOCIATE CHAIR
INSTITUTE FOR BIOENGINEERING AND BIOSCIENCE
GEORGE WOODRUFF SCHOOL OF MECHANICAL ENGINEERING
GEORGIA INSTITUTE OF TECHNOLOGY
ATLANTA, GA 30332

FIELDS, AARON J, PHD
ASSOCIATE PROFESSOR
DEPARTMENT OF ORTHOPAEDIC SURGERY
UNIVERSITY OF CALIFORNIA SAN FRANCISCO
SAN FRANCISCO, CA 94143

GOLDBERG, JUDITH D, SCD
PROFESSOR
DEPARTMENT OF POPULATION HEALTH
DIVISION OF BIOSTATISTICS
GROSSMAN SCHOOL OF MEDICINE
NEW YORK UNIVERSITY
NEW YORK, NY 10016

HERTEL, JAY N, PHD
PROFESSOR IN SPORTS MANAGEMENT AND CHAIR
DEPARTMENT OF KINESIOLOGY
UNIVERSITY OF VIRGINIA
CHARLOTTESVILLE, VA 22903

KATZ, SHARYN, MD
ASSOCIATE PROFESSOR
DEPARTMENT OF RADIOLOGY
PERELMAN SCHOOL OF MEDICINE
UNIVERSITY OF PENNSYLVANIA
PHILADELPHIA, PA 19104

KOCH, KEVIN M, PHD
PROFESSOR
DEPARTMENT OF RADIOLOGY
MEDICAL COLLEGE OF WISCONSIN
MILWAUKEE, WI 53226

KUTCH, JASON J, PHD
ASSOCIATE PROFESSOR
DEPARTMENT OF BIOMEDICAL ENGINEERING
DIVISION OF BIOKINESIOLOGY AND PHYSICAL THERAPY
UNIVERSITY OF SOUTHERN CALIFORNIA
LOS ANGELES, CA 90033

MARTUCCI, KATHERINE, PHD
ASSISTANT PROFESSOR
DEPARTMENT OF ANESTHESIOLOGY
DUKE UNIVERSITY SCHOOL OF MEDICINE
DURHAM, NC 27710

MCCULLY, KEVIN K, PHD
PROFESSOR EMERITUS
DEPARTMENT OF KINESIOLOGY
UNIVERSITY OF GEORGIA
ATHENS, GA 30602

MONROE, KATRINA S, PHD, PT
PROFESSOR OF PHYSICAL THERAPY
SCHOOL OF EXERCISE AND NUTRITIONAL SCIENCES
SAN DIEGO STATE UNIVERSITY
SAN DIEGO, CA 92182

MORASKA, ALBERT F, PHD
ASSISTANT PROFESSOR
COLLEGE OF NURSING
UNIVERSITY OF COLORADO ANSCHUTZ
AURORA, CO 80045

NORMANDIN, MARC, PHD
ASSOCIATE PROFESSOR
DEPARTMENT OF RADIOLOGY AND BIOMEDICAL IMAGING
SCHOOL OF MEDICINE
YALE UNIVERSITY
NEW HAVEN, CT 06520

PELIVANOV, IVAN, PHD
ASSOCIATE PROFESSOR
DEPARTMENT OF BIOENGINEERING
COLLEGE OF ENGINEERING
UNIVERSITY OF WASHINGTON
SEATTLE, WA 98195

ROBLYER, DARREN M, PHD
ASSOCIATE PROFESSOR
DEPARTMENT OF BIOMEDICAL ENGINEERING
BOSTON UNIVERSITY
BOSTON, MA 02215

ROYSTON, THOMAS J, PHD
PROFESSOR AND HEAD
DEPARTMENT OF BIOMEDICAL ENGINEERING
COLLEGES OF ENGINEERING AND MEDICINE
UNIVERSITY OF ILLINOIS, CHICAGO
CHICAGO, IL 60607

SHAHIDI, BAHAR, PHD, DPT
ASSOCIATE PROFESSOR
DEPARTMENT OF ORTHOPEDIC SURGERY
UNIVERSITY OF CALIFORNIA SAN DIEGO
LA JOLLA, CA 92037

SIKDAR, SIDDHARTHA, PHD
PROFESSOR
DEPARTMENT OF BIOENGINEERING
GEORGE MASON UNIVERSITY
FAIRFAX, VA 22030

SIMON, JULIANNA, PHD
ASSOCIATE PROFESSOR
DEPARTMENT OF BIOMEDICAL ENGINEERING
PENNSYLVANIA STATE UNIVERSITY
UNIVERSITY PARK, PA 16801

SINGH, JASVINDER A, MBBS, MPH
PROFESSOR
DEPARTMENT OF MEDICINE
DIVISION OF CLINICAL IMMUNOLOGY AND RHEUMATOLOGY
UNIVERSITY OF ALABAMA AT BIRMINGHAM
BIRMINGHAM, AL 35233

SINGH, VINITA, MD
ASSOCIATE PROFESSOR
DEPARTMENT OF ANESTHESIOLOGY
EMORY UNIVERSITY SCHOOL OF MEDICINE
ATLANTA, GA 30319

SNEAG, DARRYL, MD
ASSOCIATE PROFESSOR
DEPARTMENT OF RADIOLOGY
WEILL MEDICAL COLLEGE OF CORNELL UNIVERSITY
NEW YORK, NY 10065

WANG, HAO, PHD
ASSOCIATE PROFESSOR
DEPARTMENT OF ONCOLOGY
SCHOOL OF MEDICINE
JOHNS HOPKINS UNIVERSITY
BALTIMORE, MD 21205

WASAN, AJAY D, MD
PROFESSOR AND VICE CHAIR
DEPARTMENTS OF ANESTHESIOLOGY, PERIOPERATIVE
MEDICINE AND PSYCHIATRY
SCHOOL OF MEDICINE
UNIVERSITY OF PITTSBURGH
PITTSBURGH, PA 15206

WU, JIA, PHD
ASSISTANT PROFESSOR
DEPARTMENT OF IMAGING PHYSICS
DIVISION OF DIAGNOSTIC IMAGING
MD ANDERSON CANCER CENTER
HOUSTON, TX 77030

SCIENTIFIC REVIEW OFFICER

HUANG, SHIYONG, PHD
SCIENTIFIC REVIEW OFFICER
OFFICE OF SCIENTIFIC REVIEW
DIVISION OF EXTRAMURAL ACTIVITIES
NATIONAL CENTER FOR COMPLEMENTARY AND
INTEGRATIVE HEALTH, NATIONAL INSTITUTES OF HEALTH
BETHESDA, MD 20892-5475

EXTRAMURAL SUPPORT ASSISTANT

INDUSTRIOUS, GLEANZA
EXTRAMURAL SUPPORT ASSISTANT
OFFICE OF SCIENTIFIC REVIEW
DIVISION OF EXTRAMURAL ACTIVITIES
NATIONAL CENTER FOR COMPLEMENTARY AND
INTEGRATIVE HEALTH, NATIONAL INSTITUTES OF HEALTH
BETHESDA, MD 20892-5475

STINNETTE, ENDIA V
EXTRAMURAL SUPPORT ASSISTANT
OFFICE OF SCIENTIFIC REVIEW
DIVISION OF EXTRAMURAL ACTIVITIES
NATIONAL CENTER FOR COMPLEMENTARY AND
INTEGRATIVE HEALTH, NATIONAL INSTITUTES OF HEALTH
BETHESDA, MD 20892-5475

Consultants are required to absent themselves from the room during the review of any application if their presence would constitute or appear to constitute a conflict of interest.
